# Aortic Valve Replacement with a Conventional Stented Bioprosthesis *versus* Sutureless Bioprosthesis: a Study of 763 Patients

**DOI:** 10.21470/1678-9741-2017-0088

**Published:** 2018

**Authors:** Syed Saleem Mujtaba, Simon M. Ledingham, Asif Raza Shah, Thasee Pillay, Stephan Schueler, Stephen Clark

**Affiliations:** 1 Department of Cardiothoracic Surgery, Freeman Hospital, Freeman Road, United Kingdom of Great Britain and Northern Ireland.

**Keywords:** Aortic Valve/Surgery, Heart Valve Prosthesis/Utilization, Heart Valve Prosthesis Implantation

## Abstract

**Objective:**

The aim of this retrospective study was to compare early postoperative outcomes after aortic valve replacement (AVR) with sutureless bioprostheses and conventional stented bioprostheses implanted through median sternotomy.

**Methods:**

From January 2011 to December 2016, 763 patients underwent aortic valve replacement with bioprostheses; of these, 139 received a Perceval S sutureless valve (Group A) and 624 received a Perimount Magna Ease valve (Group B). These groups were further divided into A1 (isolated Perceval AVR), A2 (Perceval AVR with coronary artery bypass grafting [CABG]), B1 (isolated conventional stented bioprosthesis), and B2 (conventional stented bioprosthesis + CABG).

**Results:**

Patients in Group A were older (mean 74 years *vs.* 71 years; *P*<0.0001), predominantly women (53% *vs.* 32%; *P*<0.0001), had a higher logistic EuroSCORE (3.26 *vs.* 2.43; *P<*0.001), more preoperative atrial fibrillation (20% *vs.* 13%; *P*=0.03), and had a lower reopening rate for bleeding (2.1% *vs.* 6.7%; *P*=0.04). Compared to Group B1, Group A1 had shorter cross-clamp (mean 40 min *vs.* 57 min; *P*≤0.0001) and bypass times (mean 63 min vs. mean 80 min; *P*=0.02), and they bled less postoperatively (mean 295 ml *vs.* mean 393 ml; *P*=0.002). The mean gradient across Perceval valve was 12.5 mmHg while its effective orifice area was 1.5 cm^2^.

**Conclusion:**

In our retrospective study of 763 patients, sutureless valve group patients are older, mostly women, more symptomatic preoperatively, and have higher logistic EuroSCORE. They have shorter cross-clamp and bypass times, less postoperative bleeding, and reduced incidence of reopening. Further studies are needed to evaluate the clinical benefits in short, mid, and long-terms.

**Table:** 

Abbreviations, acronyms & symbols	
AF	= Atrial fibrillation
AVR	= Aortic valve replacement
CABG	= Coronary artery bypass grafting
CBP	= Cumulative bypass
CCC	= Cumulative cross-clamp
CPB	= Cardiopulmonary bypass
EOA	= Effective orifice area
ICU	= Intensive care unit
LVEF	= Left ventricular ejection fraction
MPG	= Mean pressure gradients
NASCA	= National Adult Cardiac Surgery Audit
NICOR	= National Institute for Cardiovascular Outcomes Research
PPM	= Patient prosthesis mismatch
SCTS	= Society for Cardiothoracic Surgeons
TAVI	= Transcatheter aortic valve implantation

## INTRODUCTION

Aortic valve replacement (AVR) is the treatment of choice for aortic valve stenosis when it is a symptomatic severe aortic stenosis (≤1 cm^2^/m^2^) or an asymptomatic with left ventricular dysfunction or combined with other cardiac surgery procedure^[1]^. Surgical AVR still represents the gold standard treatment in patients with severe aortic valve stenosis^[2]^. Owing to the increasing age of the patient population in Western world, there has been an increase in the prevalence of patients with valvular heart disease eligible for AVR^[3]^. A majority of these geriatric patients are females of small stature with corresponding small aortic roots. It has been suggested that patient prosthesis mismatch (PPM) may be associated with less regression of left ventricular hypertrophy and lower survival^[4]^. Given the increasing number of comorbidities and increasing age of patients, a tendency has emerged to use biological valve implants thus avoiding the need for long-term anticoagulation therapy. Although the concept of transcatheter aortic valve implantation (TAVI) appears attractive, the calcified aortic valve is not removed during this procedure. Therefore paravalvular leakage remains an important issue with this technique^[5]^. Other important concerns are access site-related problems and device malpositioning.

The recent introduction of sutureless bioprostheses may offer an additional tool in the therapeutic armamentarium as these valves do not need to be sutured into place, resulting in shorter cross-clamp and cardiopulmonary bypass (CPB) times which may be beneficial in older patients with comorbid conditions. Moreover, due to the absence of a sewing ring, these valves exhibit favourable hemodynamic properties. Excellent outcomes have been demonstrated with sutureless AVR in minimally invasive surgical setting^[6]^.

This study provides a comparison between the sutureless Perceval valve and the conventional stented sutured biological valve in an attempt to better define the role of sutureless AVR in the treatment of critical aortic valve stenosis.

## METHODS

This is a retrospective, observational cohort study of consecutive patients with aortic valve disease who underwent AVR with sutureless and conventional bioprostheses between January 2011 and December 2016. During this period, 763 patients underwent AVR with bioprostheses. Of these, 139 received a Perceval sutureless valve (Sorin, Saluggia, Italy) (Group A) and 624 received a Carpentier-Edwards Perimount Magna Ease aortic valve (Edwards Lifesciences, Irvine, CA, USA) (Group B). Group A was further divided into isolated Perceval valve (A1) and Perceval valve combined with coronary artery bypass grafting (CABG) (A2). Similarly, Group B was further divided into isolated Perimount magna ease (B1) and Perimount magna ease with CABG (B2). All patients were operated upon by one of six different surgeons at our institution. Preoperative characteristics and postoperative data of all these patients were studied retrospectively and Group A was compared with Group B.

If the patients had indications for AVR and wished to have a biological valve, they were given a Perceval valve, provided they had no contraindications. Although the age range of our patients for Perceval valve is 47-86 years old, very few young patients (<60 years old) had Perceval valve, as it is evident from mean and median age. Most of the young patients had a combination of bicuspid and early degenerative aortic valve stenosis. We totally respected our patient's wishes, if they preferred to have tissue valve, we provided them with Perceval valve, as long as they fulfilled the criterion.

For Perceval valve cases, congenital pure bicuspid aortic valves (Sievers type 0) with abnormal sinotubular junction (annulus ratio or aortic annulus greater than 27 mm or lesser than 19 mm) were excluded. Patients with ascending aortic aneurysm or dissection, emergency intervention, acute endocarditis, redo cases and other combined cases (beside AVR+CABG) were also excluded from both groups.

### Statistical Analysis

The data used in this analysis were extracted from National Adult Cardiac Surgery Audit (NASCA) database. This audit is managed by National Institute for Cardiovascular Outcomes Research (NICOR), with clinical direction and strategy provided by the Society for Cardiothoracic Surgeons (SCTS) and the Project Board. Analysis was performed using MS Excel. Numerical values were compared using an independent t-test, with a two-tailed distribution assuming unequal variances. Categorical variables were compared using Chi-squared χ^2^ analysis.

### Surgical Technique

After a full median sternotomy, standard CPB was established by cannulation of the ascending aorta and the right atrium. The heart was vented through the right superior pulmonary vein or the main pulmonary artery. Antegrade cold blood cardioplegia was used for myocardial protection. Continuous carbon dioxide insufflation was used routinely after sternotomy until closure of the aortotomy. In combined cases, distal end coronary anastomoses were performed before opening the aorta, and proximal end was performed after the cross-clamp is taken off, with the side biter on.

### Perceval Valve

The ascending aorta was incised transversally 1.5 cm above the sinotubular junction in order to leave a free edge for closure of the aortotomy after implantation of the device. The aortic valve was removed, and the annulus was decalcified in the usual fashion. The aortic orifice was measured with valve manufacturer's sizers.

Three 4/0 polypropylene guiding sutures were passed through the nadir of the aortic annulus. An appropriately sized prosthesis was collapsed on a side table and placed into the delivery system.

The three guiding sutures were passed through the three guides arising from the annular ring of the prosthesis, which was consequently seated on the fully debrided annulus. Once the delivery system was in position, the valve was deployed by turning the release screw and leaving the valve in place. The delivery system and guiding sutures were then removed. The field was rinsed with warm saline, and the prosthesis was dilated at four atmospheres for 30 seconds.

### Perimount Magna Valve

A transverse aortotomy was made 2 cm above the right coronary artery. Semicontinuous 2/0 Prolene sutures or interrupted non-pledgeted Ethibond sutures were used to stitch the aortic valve to the annulus. After closure of the aortotomy, transesophageal echocardiography was performed to assess the correct implantation of the prosthesis and the presence of any paravalvular leak.

## RESULTS

Preoperative patient characteristics are outlined in Tables 1 and 2.

**Table 1:** Preoperative summary.

	Group A		Group B		Chi-squared test
	n =139	%	n =624	%	*P*
Gender (M/F)	65/74	47/53	459/215	68/32	<0.0001
Procedure types					
Isolated aortic valve replacement	92	66	426	68	
AVR+CABG or other procedures	47	34	198	32	
History of cigarette smoking	83	60	410	66	0.18
History of hypertension	106	76	418	67	0.03
Renal disease at time of surgery	1	0.70	5	0.80	0.92
History of pulmonary disease, *i.e*. COPD, asthma	32	23.02	126	20.19	0.46
History of neurological disease, *i.e*. TIA, CVA	25	18	71	11	0.03
Angina status before surgery					
0. No angina	49	35	316	51	
1. No limitation of physical activity	33	24	127	20	
2. Slight limitation of ordinary activity	29	21	112	18	
3. Marked limitation of ordinary physical activity	25	18	56	9	
4. Symptoms at rest or minimal activity	3	2	13	2	
Angina symptoms	90	64.7	308	49.4	0.001
Dyspnoea status before surgery					
1. No limitation of physical activity	12	8.70	122	19.55	
2. Slight limitation of ordinary physical activity	53	38	264	42	
3. Marked limitation of ordinary physical activity	71	51	220	35	
4. Symptoms at rest or minimal activity	3	2	18	3	
Dyspnoea symptoms	127	91.4	502	80.4	0.002
History of diabetes mellitus	30	22	120	19	0.53
Preoperative heart rhythm					
0. Sinus rhythm	109	78	530	84.94	
1. Atrial fibrillation/flutter	28	20	82	13	0.03
2. Complete heart block/pacing	2	1.40	3	0	0.21
3. Other abnormal rhythm	0	0	4	1	
Ejection fraction category					
1. Good (LVEF > 50%)	111	80	498	79.8	
2. Fair (LVEF 30-50%)	22	16	85	14	0.50
3. Poor (LVEF < 30%)	6	4	40	6	0.35

AVR=aortic valve replacement; CABG=coronary artery bypass grafting; COPD=chronic obstructive pulmonary disease; CVA=cerebrovascular accident; F=female; LVEF=left ventricular ejection fraction; M=male; TIA=transient ischemic attack

**Table 2:** Preoperative summary (comparison between Groups A and B).

	Group A range	Mean	Median	Group B range	Mean	Median	*P*
Age of patients at time of procedure	47-86	74.3	75.5	34-91	71.74	73	<0.0001
Logistic EuroSCORE comparison	0.53-18.886	3.26	2.46	0-16.527	2.43	1.84	0.001
Height (cm)	140-185	162	162	138-190	166.30	167	<0.0001
Weight (kg)	40.3-158	79	74	44.9-181.6	81.47	80	0.13

There was no difference between Groups A and B in history of preoperative renal impairment or pulmonary disease. Group A had more female patients (A=53% *vs.* B=32%; *P*<0.0001) and patients with hypertension (76% *vs.* 67%; *P*=0.03) and neurological dysfunction (18% *vs.* 11%; *P*=0.03). Also, Group A had more patients with angina (90% vs. 49%; *P*=0.001), dyspnoea (91% *vs.* 80%; *P*=0.002), and preoperative atrial fibrillation (AF) (20% *vs.* 13%; *P*=0.03). In Group A, patients were older (range: 47-86 years old, mean 74 *vs.* range 34-91, mean 71; *P*=0.0001) and had a higher logistic EuroSCORE (3.26 *vs.* 2.43; *P*=0.001).

Intraoperative and early postoperative variables are summarized in Tables 3 and 4. Cross-clamp (range 21-114 min, mean 40 min *vs.* range 24-164 min, mean 57 min; *P*<0.0001) and bypass times (range 25-172 min, mean 63 min *vs.* range 19-285 min, mean 80 min; *P*=0.002) were shorter in the isolated Perceval valve group (Group A1) compared to the isolated conventional valve group (Group B1). When A2 (Perceval valve + CABG) was compared with B2 (conventional valve + CABG), A2 had a shorter cross-clamp time (range 28-127 min, mean 68 min *vs.* range 40-177, mean 78 min; *P*=0.02), but no significant difference was found in bypass time (range 38-403 min, mean 107 min *vs.* range 60-280 min, mean 112 min; *P*=0.51).

**Table 3:** Intraoperative and early postoperative summary of isolated AVR.

Intraoperative and early postoperative summary	Isolated AVR: Perceval			Isolated AVR: Perimount			Isolated valve
	Group A1 n=92			Group B1 n=426			Group A
	Range	Mean	Median	Range	Mean	Median	*P*
Cumulative cross-clamp time (min)	21-114	40	37	24-164	56.6	52	<0.0001
Cumulative bypass time	25-172	63	59	19-285	80.1	76	0.02
Postoperative blood loss at 12 hours	50-2000	295	225	100-2725	393.3	300	0.002
ICU stay in days	1-32	3.4	1	1-34	2.4	1	0.07

AVR=aortic valve replacement; ICU=intensive care unit

**Table 4:** Intraoperative and early postoperative summary of AVR+CABG.

Intraoperative and early postoperative summary	Perceval AVR+CABG			Perimount AVR+CABG			
	Group A2 n=47			Group B2 n=198			
	Range	Mean	Median	Range	Mean	Median	*P*
Cumulative cross-clamp time (min)	28-127	68	61	40-177	78.3	72	0.02
Cumulative bypass time	38-403	107	87	60-280	112.7	107	0.51
Postoperative blood loss at 12 hours	50-1200	457	400	100-2825	484.9	380	0.58
ICU stay in days	1-93	6.8	3	1-34	3.2	1	0.09

AVR=aortic valve replacement; CABG=coronary artery bypass grafting; ICU=intensive care unit

The mean gradient across Perceval valve was found to be higher for the smaller valves and lower for the larger valves. It ranges from 6-18 mmHg and its mean value was 12.5 mmHg. Similarly, its effective orifice area (EOA) varies according to the valve sizes, but its mean value was 1.5 cm^2^.

The incidence of complications over the early postoperative course is depicted in Table 5. In this study, we used first 12 hours drainage, as traditionally first 12 hours drainage is recorded in NASCA database and it is easy to retrieve. Most of the time it is the total drainage, but not always.

**Table 5:** Postoperative summary (comparison between Groups A and B).

Postoperative summary	Group A: Perceval		Group B: Perimount		
	n = 139	%	n = 624	%	*P*
Reoperation for bleeding, tamponade, or valvular problems	3	2.10%	42	6.73%	0.04
Sternal wound infection	2/139	1.43%	14	2.24%	0.55
New postoperative neurological dysfunction	4/139	2.80%	9	1.44%	0.24
New HF/dialysis postoperatively	5/139	3.60%	37	5.93%	0.28
Patient status at discharge (mortality)	3/139	2.10%	15	2.40%	0.86
SIRS	21/139	15%	111	18%	0.45
Arrhythmias					
None	88	63%	342	55%	
AF/Flutter	46	33%	258	41%	0.07
Permanent pacemaker	5	3.60%	12	1.92%	0.23

AF=atrial fibrillation; HF=haemofiltration; SIRS=systemic inflammatory response syndrome

Isolated Perceval group (Group A1) had less bleeding in first 12 hours compared to the isolated conventional valve group (Group B1) (range 50-2000 ml, mean 295 ml *vs.* range 100-2725 ml, mean 393 ml; *P*=0.002), but there was no significant difference when A2 (Perceval valve + CABG) was compared with B2 (conventional valve + CABG) (range 50-1200 ml, mean 457 ml *vs.* range 100-2825 ml, mean 485 ml; = 0.58). There were more patients reoperated for bleeding or tamponade in Group B than in Group A (2.1% *vs.* 6.7%; *P*=0.04). There was no significant difference in postoperative neurological dysfunction, renal impairment, AF, permanent pacemaker requirement, or mortality.

## DISCUSSION

New scientific and technologic achievements have allowed a continuous decrease in mortality and morbidity for AVR, in spite of the more complex and elderly patients being referred for surgery. The primary goal of AVR is to alleviate the pressure overload on the left ventricle and to allow regression as well as remodelling of the left ventricular mass. A smaller-sized prosthetic valve may result in so-called PPM.

Therefore, different options have been proposed for patients with small aortic root presenting for AVR, *i.e.*: stentless valves, aortic root enlargement, and even complete aortic root replacement. However, all these options are technically more complex and take a longer operating time. Hence, they have not been popular with surgeons.

Magovern et al.^[7]^ introduced the concept of sutureless aortic valve in the 1960s with a ball-cage-type mechanical valve for sutureless implantation. It had its own disadvantages*, i.e.*: high incidence of paravalvular leaks, bulky size and it was not suitable for small annuli^[8]^. There was a high incidence of thromboembolism (42%) and reoperation (16%). This valve continued to be used however until 1980.

The introduction of sutureless bioprostheses in the last decade offered a unique therapeutic opportunity given the possibility to potentially yield the advantages of a traditional surgical valve replacement (as the native valve can be fully excised) and a transcatheter approach (as it can be rapidly implanted). Moreover, aortic cross-clamp and total cardiopulmonary times are also reduced.

Therefore, the aim of the current study was to analyse and compare the early clinical outcomes of conventional AVR with a sutureless valve prosthesis.

Shrestha et al.^[9]^ highlighted the advantages of sutureless valves for geriatric patients with small aortic roots. They showed that Perceval sutureless valve implants are associated with shorter cross-clamp and CPB times compared to conventional biological valves, even though most of these patients were operated on via minimally invasive access.

Moreover, due to the absence of a sewing ring, these valves have favourable EOA and haemodynamics for any given size. This may potentially result in better haemodynamics even without root enlargement in small annular sizes.

Gilmanov et al.^[10]^ reviewed 515 patients undergoing primary AVR through a right anterior mini-thoracotomy (269 conventional *vs*. 246 sutureless prostheses). They showed that the use of Perceval sutureless valve had shorter cross-clamp, bypass, and mechanical ventilation times. Pollari et al.^[11]^ studied 566 patients who underwent AVR with bioprostheses. Of these, 166 received a sutureless valve and 400 received a stented valve. Aortic cross-clamp and CPB times were significantly shorter in the sutureless group. Patients from the sutureless group required blood transfusion less frequently and had shorter intensive care unit (ICU) stay, hospital stay, and intubation time.

A shorter procedural time in the sutureless group is associated with better clinical outcomes and reduced hospital costs. Dalén et al.^[12]^ compared 182 patients who underwent a ministernotomy with a sutureless bioprosthesis and 383 patients who had full sternotomy with a stented bioprosthesis. They concluded that sutureless bioprostheses were associated with shorter aortic cross-clamp and CPB times and less blood transfusion.

In our study, the Perceval valve group had lower incidence of total drainage and re-exploration rate. We think it happens because of a short bypass time. We have clearly shown that Perceval valve patients have shorter cross-clamp and bypass times and lower incidence of postoperative bleeding, but longer ICU stay (not statistically significant). We think that if these patients had had a traditional Perimount Magna Ease valve, they might have had a potentially longer ICU stay. It is only an assumption, but we need to do a randomised control trial to prove it.

Additionally, in our study, patients with isolated AVR with Perceval valve spent 3.4 days (mean) and 1 day (median) in ICU. Gilmanov et al.^[10]^ observed the same ICU stay as 1 day (median). In other studies, ICU stay was not very different, *i.e.*: Shrestha et al.^[9]^ (1.8±1.8 days), Pollari et al.^[11]^ (2±1.2 days), and Dalén et al.^[12]^ (2.4±2.4 days).

Our results correlated well with those from Shrestha et al.^[9]^ and Gilmanov et al.^[10]^, although they operated on small aortic root and through right mini-thoracotomy, respectively. Pollari et al.^[11]^ also had shorter cross-clamp and bypass times like us, but contrary to our study, their ICU stay was shorter in the Perceval group. Our results also matched those from Dalén et al.^[12]^, but they showed less blood transfusion, which we did not.

Our cumulative cross-clamp (CCC) and cumulative bypass (CBP) times for isolated AVR with Perceval valve match quite well with those from previous studies. Our mean CCC & CBP times are 40 and 63 minutes, respectively. The same times for Shrestha et al.^[9]^ were 30.1±9.0/58.7±20.9 minutes, although most of these patients were operated on via minimally invasive access. Pollari et al.^[11]^ reported CCC & CBP times of 35±12 and 71±11 minutes, respectively, but they also included some redo cases. CCC & CBP times for Dalen et al.^[12]^ were 40±15 and 69±20 minutes, respectively, although they performed their surgeries via ministernotomy. Gilmanov et al.^[10]^ implanted Perceval valve through right mini-thoracotomy and their median CCC & CBP times were 56 and 90 minutes, respectively. All these studies, including ours, showed shorter CCC & CBP times compared to conventional biological valve and this difference was statistically significant. Prolonged aortic cross-clamp time significantly correlates with major postoperative morbidity and mortality in both low- and high-risk cardiac surgery patients. This effect increases with prolonged cross-clamp time^[13]^. Al-Sarraf et al.^[13]^ have shown that by using cross-clamp time as a continuous variable, an incremental increase of 1 min interval in cross-clamp time was associated with a 2% increase in mortality in both low- and high-risk groups. In addition, high-risk patient populations such as those with diabetes or depressed left ventricular ejection fraction (LVEF) were found to benefit the most from a reduction in aortic cross-clamp time^[14]^.

Our mean pressure gradients (MPG) and EOA were comparable with the values reported in literature for Perceval valves. Folliguet et al.^[15]^ (10.4±4.3 mmHg), Santarpino et al.^[16]^ (13.4±2.8 mmHg), Flameng et al.^[17]^ (11 range 5-28 mmHg), D'Onofrio et al.^[18]^ (10.95±3.72 mmHg), and the Cavalier Trial^[19]^ (10.24 mmHg) reported pre-discharge MPG, while Shrestha et al.^[9]^ discussed 12-month follow-up data (10±5 mmHg). Our EOA was also compatible with the Cavalier study results^[19]^ (1.46 cm^2^).

In our study, the Perceval group includes older patients, more women, and more comorbidities, as evident by a higher logistic EuroSCORE. Although their postoperative drainage is lower, because of more comorbidities and for being sicker, they spent more time in ICU, but this difference was not statistically significant. Moreover, higher logistic EuroSCORE was not reflected in a higher mortality, as the difference was not statistically significant. Maybe the factor of a higher comorbidity was buffered by a shorter cross-clamp time.

### Limitations

This study carries all the limitations that a retrospective analysis design implies. Patients in the sutureless group were operated upon more recently than the majority of those receiving conventional valves. Propensity matching is not performed which could have provided more accurate comparison.

## CONCLUSION

Despite the limits of its retrospective design, this study represents the largest single-center comparison between Perceval sutureless valve and conventional bioprostheses for AVR. We observed that in our study of 763 patients, sutureless valve group patients are older, mostly women, more symptomatic, and have higher logistic EuroSCORE. Despite this, those operated on with the Perceval valve showed shorter cross-clamp and bypass times, less postoperative bleeding, and a reduced incidence of reopening. Further studies are needed to evaluate the clinical benefits in short, mid and long-terms.

**Table:** 

Authors' roles & responsibilities	
SSM	Substantial contributions to the conception or design of the work; or the acquisition, analysis, or interpretation of data for the work; final approval of the version to be published
SML	Substantial contributions to the conception or design of the work; or the acquisition, analysis, or interpretation of data for the work; final approval of the version to be published
ARS	Substantial contributions to the conception or design of the work; or the acquisition, analysis, or interpretation of data for the work; final approval of the version to be published
TP	Substantial contributions to the conception or design of the work; or the acquisition, analysis, or interpretation of data for the work; final approval of the version to be published
SS	Substantial contributions to the conception or design of the work; or the acquisition, analysis, or interpretation of data for the work; final approval of the version to be published
SC	Substantial contributions to the conception or design of the work; or the acquisition, analysis, or interpretation of data for the work; final approval of the version to be published

## References

[1] Nishimura RA, Otto CM, Bonow RO, Carabello BA, Erwin JP 3rd, Guyton RA, ACC/AHA Task Force Members (2014). 2014 AHA/ACC Guidelines for the management of patients with
valvular heart disease: executive summary: a report of the American College
of Cardiology/American Heart Association Task Force on Practice
Guidelines. Circulation.

[2] Vahanian A, Alfieri O, Andreotti F, Antunes MJ, Barón-Esquivias G, Baumgartner H, ESC Committee for Practice Guidelines (CPG), Joint Task Force on the Management of Valvular Heart Disease of the
European Society of Cardiology (ESC), European Association for Cardio-Thoracic Surgery (EACTS) (2012). Guidelines on the management of valvular heart disease (version
2012): the Joint Task Force on the Management of Valvular Heart Disease of
the European Society of Cardiology (ESC) and the European Association for
Cardio-Thoracic Surgery (EACTS). Eur J Cardiothorac Surg.

[3] Bridgewater B, Gummert J, Kinsman R, Walton P (2010). Towards global benchmarking: the Fourth EACTS Adult Cardiac Surgical
Database Report.

[4] Mohty D, Dumesnil JG, Echahidi N, Mathieu P, Dagenais F, Voisine P (2009). Impact of prosthesis-patient mismatch on long-term survival after
aortic valve replacement: influence of age, obesity, and left ventricular
dysfunction. J Am Coll Cardiol.

[5] Martens S, Sadowski J, Eckstein FS, Bartus K, Kapelak B, Sievers HH (2011). Clinical experience with the ATS 3f Enable® sutureless
bioprosthesis. Eur J Cardiothorac Surg.

[6] Santarpino G, Pfeiffer S, Concistrè G, Fischlein T (2012). Perceval S aortic valve implantation in mini-invasive surgery:
the simple sutureless solution. Interact Cardiovasc Thorac Surg.

[7] Magovern GJ, Cromie HW (1963). Sutureless prosthetic heart valves. J Thorac Cardiovasc Surg.

[8] Scott SM, Sethi GK, Flye MW, Takaro T (1976). The sutureless aortic valve prosthesis: experience with and
technical considerations for replacement of the early model. Ann Surg.

[9] Shrestha M, Maeding I, Höffler K, Koigeldiyev N, Marsch G, Siemeni T (2013). Aortic valve replacement in geriatric patients with small aortic
roots: are sutureless valves the future?. Interact Cardiovasc Thorac Surg.

[10] Gilmanov D, Miceli A, Ferrarini M, Farneti P, Murzi M, Solinas M (2014). Aortic valve replacement through right anterior minithoracotomy:
can sutureless technology improve clinical outcomes?. Ann Thorac Surg.

[11] Pollari F, Santarpino G, Dell'Aquila AM, Gazdag L, Alnahas H, Vogt F (2014). Better short-term outcome by using sutureless valves: a
propensity-matched score analysis. Ann Thorac Surg.

[12] Dalén M, Biancari F, Rubino AS, Santarpino G, Glaser N, De Praetere H (2016). Aortic valve replacement through full sternotomy with a stented
bioprosthesis versus minimally invasive sternotomy with a sutureless
bioprosthesis. Eur J Cardiothorac Surg.

[13] Al-Sarraf N, Thalib L, Hughes A, Houlihan M, Tolan M, Young V (2011). Cross-clamp time is an independent predictor of mortality and
morbidity in low- and high-risk cardiac patients. Int J Surg.

[14] Ranucci M, Frigiola A, Menicanti L, Castelvecchio S, Vincentiis C, Pistuddi V (2012). Aortic cross-clamp time, new prostheses, and outcome in aortic
valve replacement. J Heart Valve Dis.

[15] Folliguet TA, Laborde F, Zannis K, Ghorayeb G, Haverich A, Shrestha M (2012). Sutureless perceval aortic valve replacement: results of two
European centers. Ann Thorac Surg.

[16] Santarpino G, Pfeiffer S, Schmidt J, Concistrè G, Fischlein T (2012). Sutureless aortic valve replacement: first-year single-center
experience. Ann Thorac Surg.

[17] Flameng W, Herregods MC, Hermans H, Van der Mieren G, Vercalsteren M, Poortmans G (2011). Effect of sutureless implantation of the Perceval S aortic valve
bioprosthesis on intraoperative and early postoperative
outcomes. J Thorac Cardiovasc Surg.

[18] D'Onofrio A, Messina A, Lorusso R, Alfieri OR, Fusari M, Rubino P (2012). Sutureless aortic valve replacement as an alternative treatment
for patients belonging to the "gray zone" between transcatheter aortic valve
implantation and conventional surgery: a propensity-matched, multicenter
analysis. J Thorac Cardiovasc Surg.

[19] Laborde F, Fischlein T, Hakim-Meibodi K, Misfeld M, Carrel T, Zembala M, Cavalier Trial Investigators (2016). Clinical and haemodynamic outcomes in 658 patients receiving the
Perceval sutureless aortic valve: early results from a prospective European
multicentre study (the Cavalier Trial). Eur J Cardiothorac Surg.

